# Role of Posterior Acoustic Shadow Width in Ultrasound in Determining Stone Size in Urolithiasis

**DOI:** 10.7759/cureus.49254

**Published:** 2023-11-22

**Authors:** Mohamed Javid, Ananda Kumar Ilangovan, Ramesh Ganapathy, Senthilkumar Sivalingam, Sudhakaran Selvaraj, Srikala Prasad, Prabhu Elumalai

**Affiliations:** 1 Urology, Chengalpattu Medical College, Chengalpattu, IND

**Keywords:** stone size, computed tomography, posterior acoustic shadow, ultrasound, urolithiasis

## Abstract

Introduction

Ultrasound (US) is frequently the initial diagnostic tool for urolithiasis, though computed tomography (CT) remains the imaging modality of choice. However, due to potential overestimations, the accuracy of US in gauging stone size has been a point of contention. This study aims to compare the accuracy of stone size measurements in US, specifically evaluating the utility of the posterior acoustic shadow (PAS) width, against the CT measurements.

Methods

We conducted a cross-sectional study where 120 adult patients (aged >18 years) with confirmed urolithiasis through CT participated. Stone sizes were assessed via both CT and US, with the PAS width also being measured in the latter. Statistical analysis compared stone size discrepancies between both CT and US measurement techniques.

Results

The study enrolled 73 males and 47 females with various stone locations. The average stone sizes were 15.93 ± 4.59 mm (CT), 18.60 ± 4.80 mm (US), and 16.69 ± 4.61 mm (PAS width). There was a mean difference of 2.67 mm (p < 0.0001) between CT and US sizes, whereas the difference between the PAS width and CT sizes was only 0.75 mm (p* *= 0.203). Stone size miscalculation by US was 16.77%, whereas it was only 4.77% for PAS width.

Conclusion

US tends to significantly overestimate stone size when compared to CT. Conversely, the measurement of the PAS width in US presents a more aligned estimation to CT outputs. Integrating PAS width into routine US reporting can enhance the accuracy of stone size estimation, optimizing urolithiasis management and patient counseling.

## Introduction

The accurate measurement of stone size is an important factor in the assessment, treatment, and follow-up of urolithiasis patients, highlighting the significance of precision in the evaluation of urolithiasis [1-4]. Although computed tomography (CT) is widely recognized as the standard imaging modality of choice for urolithiasis, ultrasound (US) is commonly employed as the initial imaging in the diagnostic and management pathway [1-3,5,6]. This can be attributed to various advantages that are exhibited by the US, such as its widespread availability, portability, cost-effectiveness, and lack of ionizing radiation [1-3,6,7]. However, previous research has revealed that the US may overestimate stone size, potentially leading to inaccurate counseling in over 20% of patients when relying on US stone size measurements alone [8-10]. This underlines a lacuna and an existing need for the exploration and development of additional tools within the US framework to enhance the precision of stone size estimation [9,10]. In US examinations, urolithiasis manifests as a hyperechoic signal accompanied by a distinct posterior acoustic shadow (PAS) positioned behind the stone [1,5]. While the hyperechoic signal boundaries may be significantly influenced by variations in US imaging modality and system settings, the characteristics of the PAS generally remain predominantly consistent [1]. Due to the paucity of existing research within this domain, our study was designed to elucidate the utility of the PAS in US examinations of urolithiasis. Our study focused on analyzing the stone size as measured directly via the US, including the size of the PAS, and comparing these dimensions with the corresponding stone size measurements obtained through CT in urolithiasis patients.

## Materials and methods

Study design and participants

Our research adopted a cross-sectional study design enrolling 120 adult patients (aged 18 years and above), each of whom was confirmed to have urolithiasis as evidenced by a CT scan. We included patients who had a diagnosis of renal pelvic calculus, renal calculus, renal pelviureteric junction (PUJ), and ureterovesical junction (UVJ) calculus. Vesical calculus was deliberately not included in the study to mitigate any undesirable effects that might arise from positional variations in size. The study was approved by the Institutional Ethics Committee of the Chengalpattu Medical College, Chengalpattu, India, ensuring adherence to ethical guidelines and the safeguarding of participants’ rights (Ref no: CMCH-21-PR-275). Informed consent was obtained from all participants when they were enrolled. 

Measurement of stone size and PAS

The diagnosis of urolithiasis was ascertained through CT, in which the length, width, and height of the stone were assessed across the axial, coronal, and sagittal sections. Among these, the dimension that exhibited the largest measurement served as the reference stone size within the CT context. The CT was performed using an Aquilion Lightning TSX035A 16-slice CT scanner.

Concurrently, a US was performed either on the same day as the CT or within a 24-hour interval, by an independent clinician who had neither prior knowledge of the CT-derived stone size nor any clinical information about the patient. In the US examination, the greatest stone size identified in any dimension was designated as the reference stone size in the US analysis. The US was conducted using a Mindray DP-50 machine.

Additionally, utilizing digital calipers integrated within the US machine, the PAS that was observed behind the stone was carefully measured at a distance of 1 cm posterior to the hyperechoic signal representing the stone. This distance was adhered to based on the precedent established by Dai et al. and Dunmire et al., who successfully employed it as a reference in their research [1,5,6]. In the absence of a discernible PAS, this was appropriately noted, and the image was not included in the study.

To elucidate the correlations and discrepancies between these imaging techniques, a comparison of the stone sizes as measured by CT and US, inclusive of an evaluation of the PAS width, was performed.

Statistical analysis

The collected data were assiduously recorded in a Microsoft Excel spreadsheet and subjected to statistical evaluation using the IBM SPSS Statistics, version 27.0 (IBM Corp., Armonk, NY). Categorical and quantitative variables were analyzed using descriptive statistics (including means, standard deviations, frequencies, and percentages). To analyze the data, descriptive statistics, including frequency and percentage analysis, were employed for categorical variables, while the mean and standard deviation (SD) were calculated for continuous variables. A t-test was conducted to examine potential differences in size measurements between US and CT as well as PAS and CT. In the above statistical tool, the probability value of 0.05 was considered significant.

## Results

Out of the 120 patients in our study, a total of 73 (60.83%) male and 47 (39.17%) female patients were included. The stone locations that were studied were as follows: renal pelvic calculus, 16 (13.33%); PUJ calculus, 36 (30%); renal calculus, 31 (25.83%); and VUJ calculus, 37 (30.83%). In all, 65 (54.17%) stones on the right side and 55 (45.83%) stones on the left side were studied.

The average stone size recorded was 15.93 ± 4.59 mm on CT, 18.60 ± 4.80 mm on US, and 16.69 ± 4.61 mm by PAS width.

The absolute mean difference between CT size and US size was 2.67 mm (p < 0.0001) (Table 1). In contrast, the absolute mean difference between CT size and PAS size was 0.75 mm, which was not statistically significant (p = 0.203) (Table 2). It was also found that the miscalculation of the stone size by the US was 16.77%, whereas it is 4.77% in PAS width.

**Table 1:** Mean difference between CT size and US size US, stone size measured by ultrasound; CT, stone size measured by computed tomography

Investigation	N	Mean	SD	T score	p-value
US (in mm)	120	18.60	4.80	4.402	0.0001
CT (in mm)	120	15.93	4.59		

**Table 2:** Mean difference between CT size and PAS size CT, stone size measured by computed tomography; PAS, measured posterior acoustic shadow width

Investigation	N	Mean	SD	T score	p-value
PAS (in mm)	120	16.69	4.61	1.276	0.203
CT (in mm)	120	15.93	4.59		

## Discussion

The historical precedence of US in nephrolithiasis management traces back to 1961 when amplitude (A)-mode sonography for renal stone localization was first utilized [1,5,6]. Propelled by contemporary concerns regarding the potential long-term detrimental ramifications of ionizing radiation exposure from CT have catalyzed a renewed interest in propelling the US more in urolithiasis management [5]. It is imperative to make an accurate estimation of the stone size as it serves as a critical determinant in evaluating the likelihood of spontaneous passage and in devising the optimal surgical interventions. Furthermore, sole reliance on the US for stone sizing has led to miscounseling in approximately 22% of clinical cases [5]. Furthermore, US has been considered inferior to CT in determining the stone size [9-12]. The two main challenges that hinder the US from getting enrolled in more widespread utilization are adequacy in detecting the stone and the accuracy in measuring the stone size [5]. With the continuous evolution and the recent advancements in diagnostic US techniques for stone detection and sizing, the US has considerably amplified its potential in the therapeutic landscape of stone disease [5]. Emerging advancements in US techniques, such as twinkling, S-mode, and 3D US, offer innovative avenues for stone detection and sizing [5]. Cumulative evidence from both phantom and human studies has indicated that measuring the width of PASs could substantially refine stone size measurement accuracy in the US [1,5,6]. So, PAS can elegantly navigate the compromise between size accuracy and radiation minimization. This can potentially generate a treatment pathway where small stones that might pass out spontaneously can be reliably identified by the US, and a CT scan may be reserved only for equivocal cases. This strategy might become more productive, especially in children and pregnant women, where there are concerns about radiation exposure [1].

In the seminal work by Dunmire et al. [6], it was discerned that the stone size estimation through US surpassed the measurements drawn from CT by an average margin of around 1.5-2 mm [13,14]. Our research extends these findings revealing an average overestimation of approximately 2.6 mm when US-derived measurements were juxtaposed with those of CT. 

Kishore and colleagues illustrated that by estimating the width of the PAS, the precision of ultrasonography in measuring stone size can be appreciably augmented and approached with an accuracy of 1 mm to that of the CT measurements [1,15]. Corroborating these findings, our analysis demonstrated a narrow divergence of 0.75 mm between PAS shadow width and CT size measurements. This demonstrates credence to the prospect of utilizing PAS width as a reliable adjunct in determining the precise stone size [1].

Our analytical comparison revealed a mean absolute difference of 2.67 mm between CT and US size (p < 0.0001). This was in contrast with a mere 0.75 mm difference between CT and PAS size (p = 0.203). These findings can be interpreted as evidence of the statistically significant overestimation of stone size by US relative to CT, whereas such overestimation by PAS width remained statistically insignificant. This leads to the understanding that PAS width emerges as a more accurate correlate to actual stone size compared to direct measurements of the hyperechogenic stone shadow in the US. This distinct insight could propel future research and clinical practice in the direction of more accurate and non-invasive methodologies in urolithiasis management. Our study has a few limitations such as the relatively small sample size and its execution within a single center. Hence, future research should be conducted on a larger scale with a more extensive patient base and should adopt a multicentric approach.

## Conclusions

From our study, it can be concluded that the US overestimates stone size significantly in comparison with CT, whereas the measurement of the PAS width gives a more accurate estimation of the true stone size than the direct stone measurement in the US. Hence, PAS width can be routinely incorporated in US reports as it is a more reliable measurement of the actual stone size, and this will help in the management and follow-up of patients with urolithiasis.
